# UNMET NEEDS & AVOIDABLE NURSING HOME PLACEMENTS AMONG BLACK, LATINO, AND WHITE PEOPLE LIVING WITH DEMENTIA

**DOI:** 10.1093/geroni/igae098.4305

**Published:** 2024-12-31

**Authors:** Jasmine Travers, Shivani Shenoy, Marissa Bergh, Aasha Raval, Andreina Jimenez

**Affiliations:** New York University, New York, New York, United States; New York University, New York, New York, United States; New York University, Long Island City, New York, United States; New York University, New York, New York, United States; New York University, New York, New York, United States

## Abstract

Many nursing home (NH) placements are avoidable considering equivalent care can be provided in the community setting. Preventing avoidable NH placements and enabling aging in the home and community has been prioritized by federal, state, and local entities. Guided by Diwan and Moriarty’s conceptual frame for Identifying Unmet Healthcare Needs of Community Dwelling Older Adults, this qualitative descriptive study explored the unmet needs and related barriers that drive avoidable NH placements among Black, Hispanic/Latino, and White people living with dementia (PLWD). One-on-one semi-structured interviews were conducted March-November 2022. Participants including PLWD, family care partners (FCP), and NH staff, were recruited from two New York NHs. Policy experts were recruited from aging organizations across the country. Data were analyzed using a directed content and thematic analysis approach. Sixty-one individuals were interviewed, 19 PLWD, 17 FCP, 15 NH staff, and 10 aging policy experts. Seven distinct themes specific to unmet needs emerged from the data and included 1) support with activities of daily living and basic home maintenance; 2) resources and services; 3) treatment; 4) socialization; 5) individual preferences; 6) function of the home; and 7) equipped, available, and supported family. Related barriers that contribute to unmet needs defined within our guiding framework included awareness, knowledge, availability, affordability, and acceptability of resources and services. Participants described multifaceted factors that contribute to the unmet needs driving avoidable NH placements. Strategies to address these unmet needs and improve the understanding of the complex home and community-based services landscape may help reduce these placements.

